# The dark side of DNA repair

**DOI:** 10.7554/eLife.03068

**Published:** 2014-05-20

**Authors:** Samuel H Wilson

**Affiliations:** 1 **Samuel H Wilson** is in the Laboratory of Structural Biology, National Institute of Environmental Health Sciences, National Institutes of Health, Research Triangle Park, United Stateswilson5@niehs.nih.gov

**Keywords:** DNA repair, mutagenesis, APOBEC deaminase, mutations, Human

## Abstract

The BER pathway is widely used to repair DNA damage in cells, but it can also introduce unwanted mutations and is sometimes hijacked by other pathways.

**Related research article** Chen J, Miller BF, Furano AV. 2014. Repair of naturally occurring mismatches can induce mutations in flanking DNA. *eLife* **3**:e02001. doi: 10.7554/eLife.02001

**Image** Deliberately introducing lesions into circular DNA molecules called episomes allows DNA repair pathways to be studied in detail

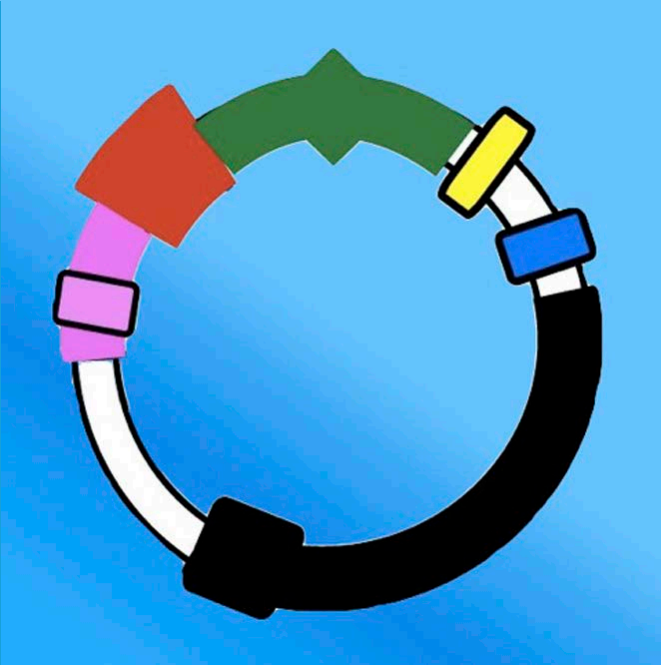

DNA can be damaged in many ways—bases can, for example, be oxidized, alkylated or deaminated—so cells need a way to repair DNA damage. Sometimes it is possible to reverse the process that caused these ‘base lesions’, but cells tend to employ a more complex approach that is called the base excision repair (BER) pathway. This process involves three basic steps: first, enzymes called nucleases break the DNA strand that is damaged on either side of the lesion so that the section containing the lesion can be removed; second, DNA polymerases make a new stretch of DNA to replace the section that has been removed, using the undamaged strand as a template; third, DNA ligases join this new stretch to the existing DNA.

However, there is a ‘dark side’ to DNA repair because the process by which the replacement DNA is synthesized is inherently error-prone. Moreover, DNA is normally tightly packaged inside the nucleus to protect it from damage: however, the BER pathway involves regions of DNA being unpackaged, and this provides other enzymes and repair pathways with access to the DNA, which can lead to lesions and mutations. The overall result is that DNA repair can sometimes lead to more rather than less damage and, in some cases, this damage can ultimately kill the cell (Fu et al., 2012).

Large-scale DNA sequencing has revealed a complex pattern of lesions and mutations that includes mutational hotspots and mutations that are linked to specific sequences of bases. In some cases the mutations appear to be linked to enzymes belonging to the APOBEC family—which change the cytosine bases in DNA into uracil bases—being able to access the DNA (Roberts et al., 2013; Nik-Zainal et al., 2014). Finally, some seemingly random mutations occur with high frequency.

Now, in *eLife*, Jia Chen, Brendan Miller and Anthony Furano—who all are based at the National Institute of Diabetes and Digestive and Kidney Diseases—have used DNA sequencing to explore the accuracy of the BER pathway in mammalian cells (Chen et al., 2014). They used genetic techniques to deliberately introduce lesions associated with different sub-pathways of the BER pathway. These lesions included: a simple strand break between two neighbouring bases (which is known as a nick); replacing the cytosine base opposite a guanine base with an abasic site; and various types of base mismatches (such as thymine/guanine, uracil/guanine or hydroxymethyl-uracil/guanine instead of cytosine/guanine). The lesions were individually engineered into episomal DNA, and the episomes were allowed to undergo repair in mammalian cells and then isolated. After passage through *E. coli*, mutants were selected, and the DNA was subjected to sequence analysis.

As expected, most of the lesions were repaired by the BER pathway (Figure 1), but there were interesting differences in the repair of the different types of lesions. In some cases, for example, there were mutations in the template strand, rather than the strand containing the lesion, which suggests that various enzymes had been able to access the template strand during the repair process.

**Figure 1.**
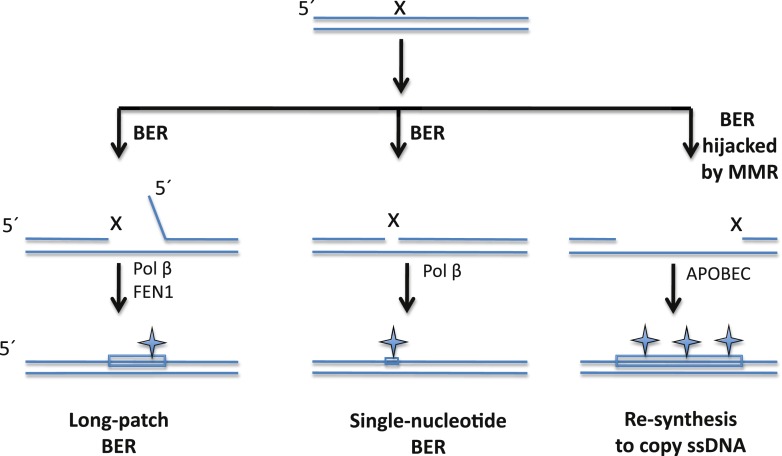
The BER pathway can repair DNA damage, but it can also induce mutations. Cells rely on the base excision repair (BER) pathway to repair base lesions in DNA. A single lesion (X) in one of the strands in double-stranded DNA can be repaired by removing the damaged base (and some neighbouring bases) and replacing it with a new stretch of DNA (shown by a rectangle): this process is called long-patch BER (left). However, the process used to synthesize the new DNA is prone to error, which can lead to the introduction of mutations (symbol). FEN1 is the nuclease that removes the flap of single-stranded DNA (ssDNA); Pol ß is the polymerase that synthesises the new DNA. It is also possible to replace just the damaged base (middle), but this single-nucleotide BER pathway can also introduce mutations. Sometimes, however, the BER pathway is hijacked by a DNA repair pathway called the mismatch repair (MMR) system that exposes a stretch of ssDNA on the 5′side of the lesion: this ssDNA then becomes a substrate for APOBEC enzymes that remove amine groups from cytosine bases, which introduces even more mutations (right). Note: figure not drawn to scale; the orientation of strands is shown.

Moreover, mutations were observed in the bases on one side of the base lesion (distal to the lesion). These ‘flanking mutations’ were shown to depend on the initial steps of the BER pathway in most cases. A number of enzymes are known to be capable of exposing single-stranded DNA on either side of a nick, but it seems surprising that the molecular machinery responsible for the BER pathway allowed these enzymes to have access to the lesions. Previously it was thought that each enzyme in the BER pathway ‘handed over’ to the next enzyme in a highly coordinated process that left little scope for third parties to have access to the DNA (Prasad et al., 2010), but the presence of the flanking mutations suggests that the lesion recognition and hand-over process is not always able to keep third parties away from the lesion.

So what actually causes the flanking mutations? Chen, Miller and Furano suggest that the BER pathway is ‘hijacked’ by another DNA repair pathway called the mismatch repair system (which, as its name suggests, repairs base mismatches). They provided evidence for this by showing that depleting factors involved in the mismatch repair system results in a reduction in flanking mutations.

Chen et al. also noticed that many of the flanking mutations involved cytosine bases that were preceded by thymine bases, with the cytosine bases changing to uracil bases. This pattern suggested that certain APOBEC enzymes—those that have a preference for the TpC sequence in single-stranded DNA—were involved. To test this idea Chen et al. depleted the level of APOBEC enzymes and, as they predicted, there was a drop in the number of flanking mutations. Taken together, the results point to the idea that the mismatch repair machinery exposes single-stranded DNA that serves as a substrate for the APOBEC enzymes, which remove amine groups from the cytosine bases to leave uracil bases (Roberts et al., 2012, 2013). DNA synthesis to copy the single-stranded DNA then leads to mutations.

Overall, the results reported by Chen, Miller and Furano open up a whole new range of questions about the introduction of mutations during DNA repair.
